# Resting‐state functional connectivity correlates of cognitive function in Mild Cognitive Impairment on functional Near Infrared Spectroscopy

**DOI:** 10.1002/alz.093543

**Published:** 2025-01-09

**Authors:** Subhashini K Rangarajan, Sonika Nichenametla, Vanteemar S Sreeraj, Vijaykumar Harbishettar, Preeti Sinha, Palanimuthu Thangaraju Sivakumar, Ganesan Venkatasubramanian

**Affiliations:** ^1^ Multimodal Brain Image Analysis Laboratory, National Institute of Mental Health and Neurosciences (NIMHANS), Bengaluru, Karnataka India; ^2^ National Institute of Mental Health and Neurosciences, Bengaluru, Karnataka India; ^3^ Translational Psychiatry Laboratory, National Institute of Mental Health and Neurosciences (NIMHANS), Bengaluru India; ^4^ National Institute of Mental Health and Neurosciences, Bangalore, Karnataka India; ^5^ Department of Psychiatry, National Institute of Mental Health and Neurosciences (NIMHANS), Bengaluru, Karnataka India; ^6^ National Institute of Mental Health and Neurosciences (NIMHANS), Bengaluru, India), Bengaluru India; ^7^ Translational Psychiatry Laboratory, National Institute of Mental Health and Neurosciences (NIMHANS), Bengaluru, Karnataka India

## Abstract

**Background:**

Mild cognitive impairment(MCI) is characterized by an impairment in one or more cognitive domains greater than expected for a person’s age and educational background with preserved functional independence. Functional Near Infra‐Red Spectroscopy (fNIRS) is a modality of non‐invasive neuroimaging that utilizes the optical properties of oxygenated and deoxygenated hemoglobin in the tissue to measure their absolute or relative concentrations following neuronal activity. fNIRS has been used to evaluate neurohemodynamics in MCI. Lower tissue oxygenation in bilateral frontal and parietal regions was found at resting state, which correlated with cognitive functioning in MCI. The objective of this study was to evaluate the correlation between cognitive functioning and resting state functional connectivity on fNIRS in MCI.

**Methods:**

The study was conducted at the Geriatric Clinic and Services, Department of Psychiatry, National Institute of Mental Health and Neurosciences (NIMHANS), Bangalore, India (Institutional Ethics Committee approval no.: NIMHANS/34th IEC (BEH.SC.DIV.)/2022) on consenting participants with MCI (N = 38). Detailed cognitive assessment was performed using a validated comprehensive computerised cognitive battery. fNIRS data acquisition was done using the NIRScout device, NIRx medical technologies LLC, CA, USA, operating on continuous wave domain, with two wavelengths of 760nm and 850 nm. 8 sources and 8 detectors were placed in a bilateral prefrontal montage placement resulting in 18 channels (9 on each side). Nodal metrics such as clustering coefficient, degree centrality, shortest path length, local efficiency and a Global metric‐ small worldness was evaluated. Correlation coefficients were computed for the nodal and global metrics with cognitive domains such as reaction time, complex attention, memory, language functioning and perceptuomotor functioning.

**Results:**

Mean age of participants was 66.18 ± 6.18 years. The mean duration of illness was 2.63 ± 2.5 years. The results of the resting state nodal and global metrics and their correlation with cognitive functioning will be presented during the conference.

**Conclusion:**

fNIRS is reported to have a temporal resolution that is comparable to fMRI, at the same time being less expensive, portable and having less interference because of motion artifacts. Findings from this study will provide insights on the functional connectivity correlates of cognitive functioning in MCI.

